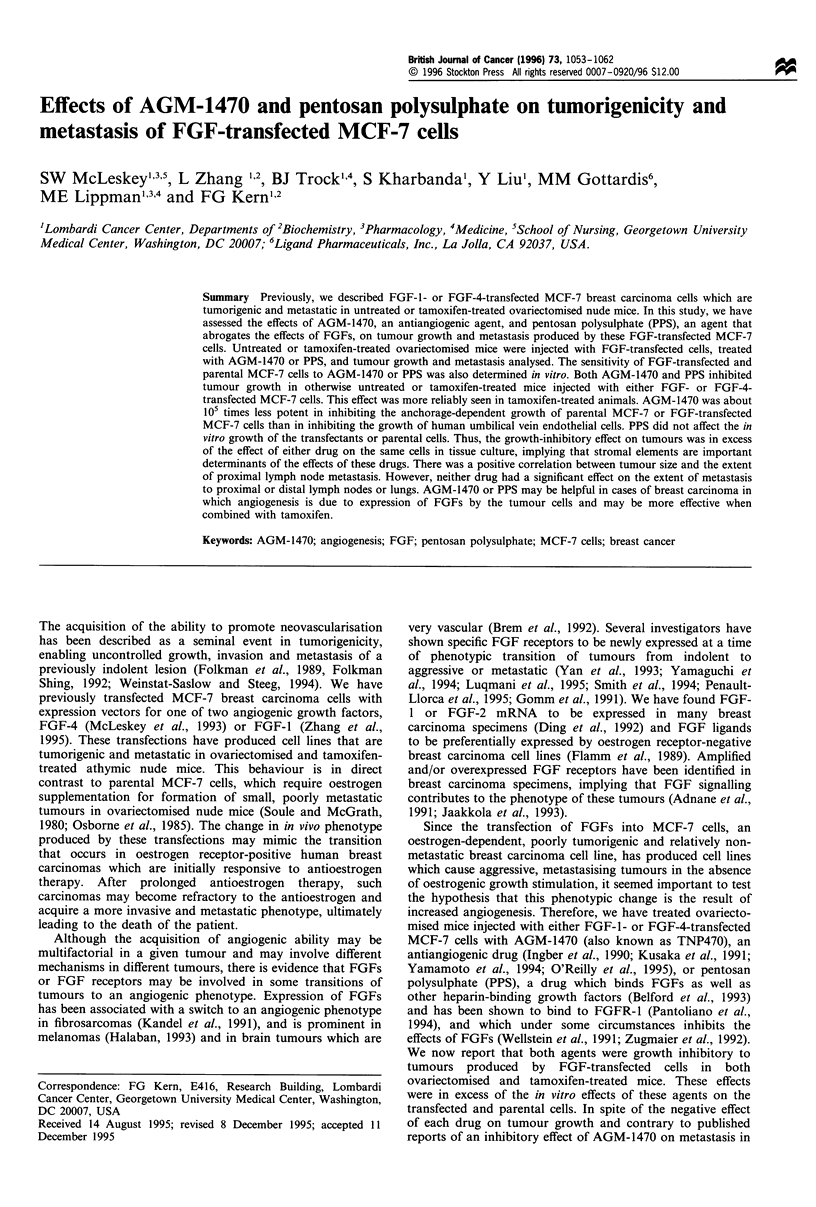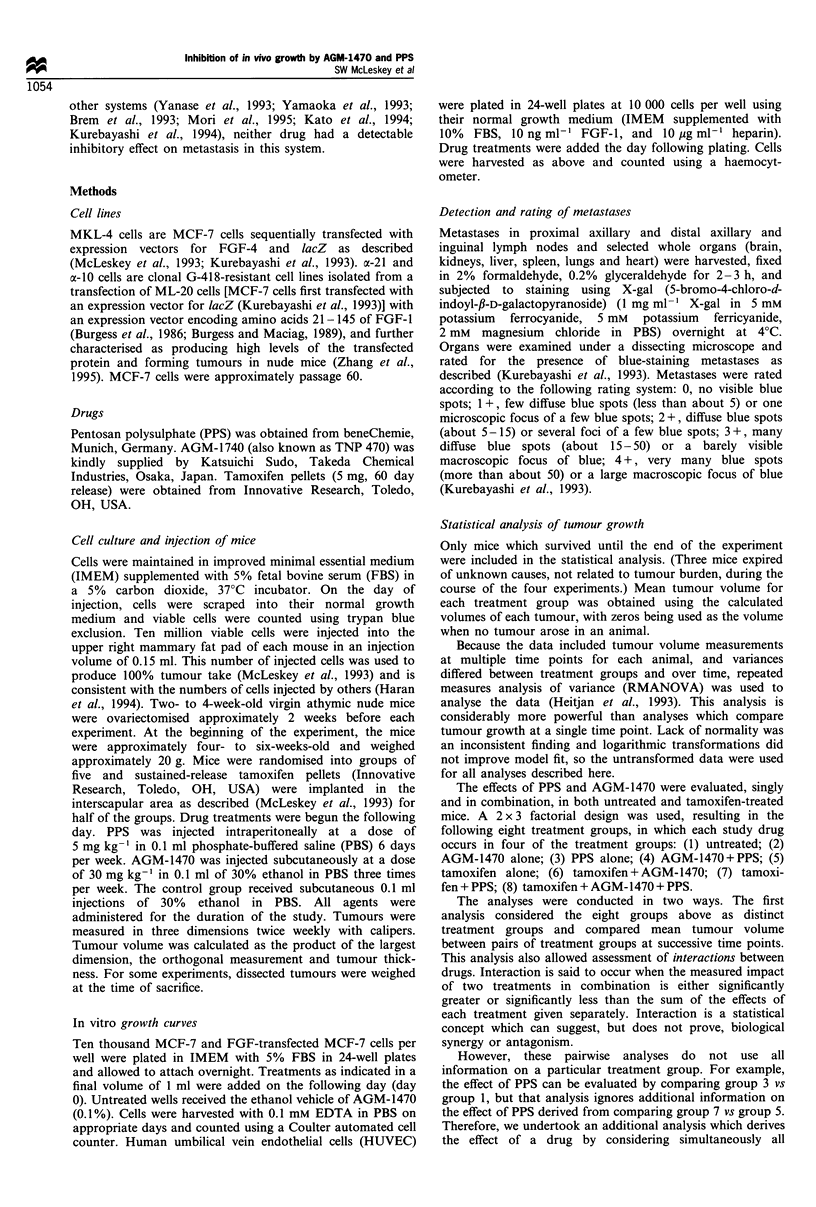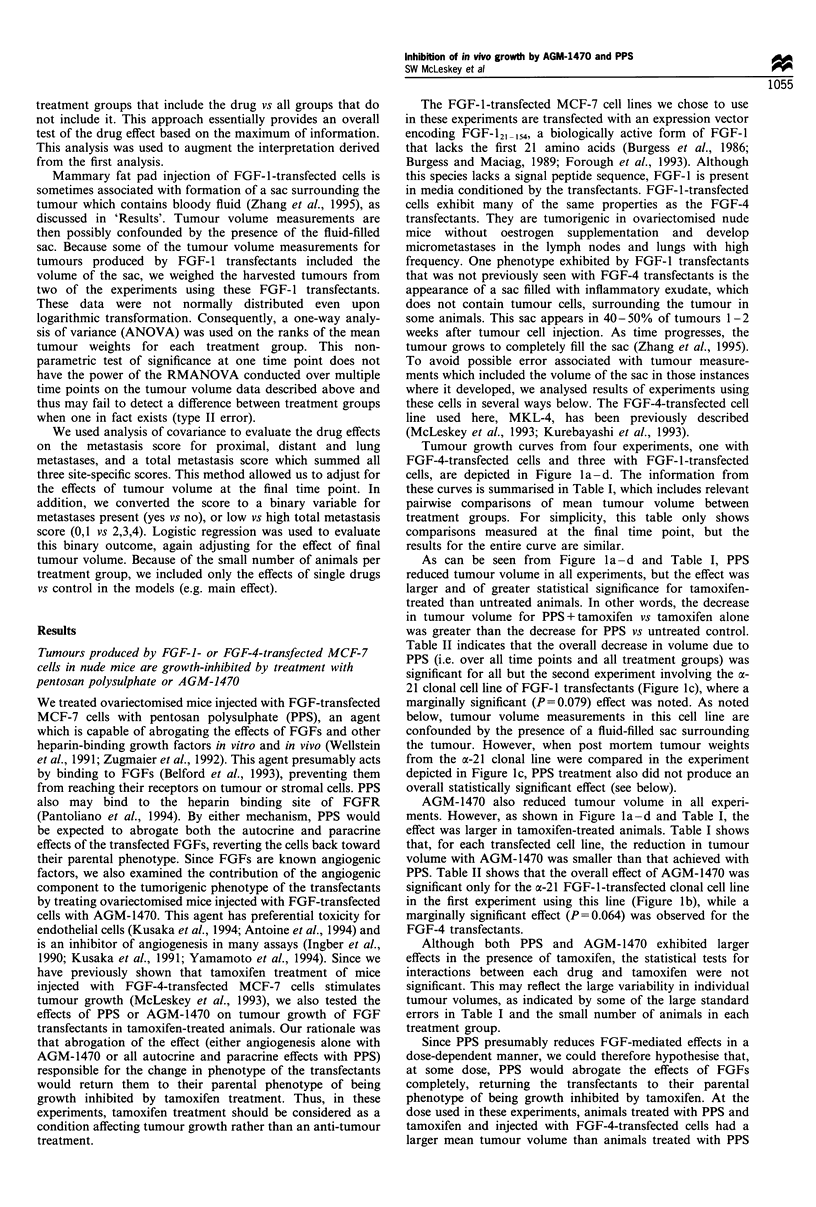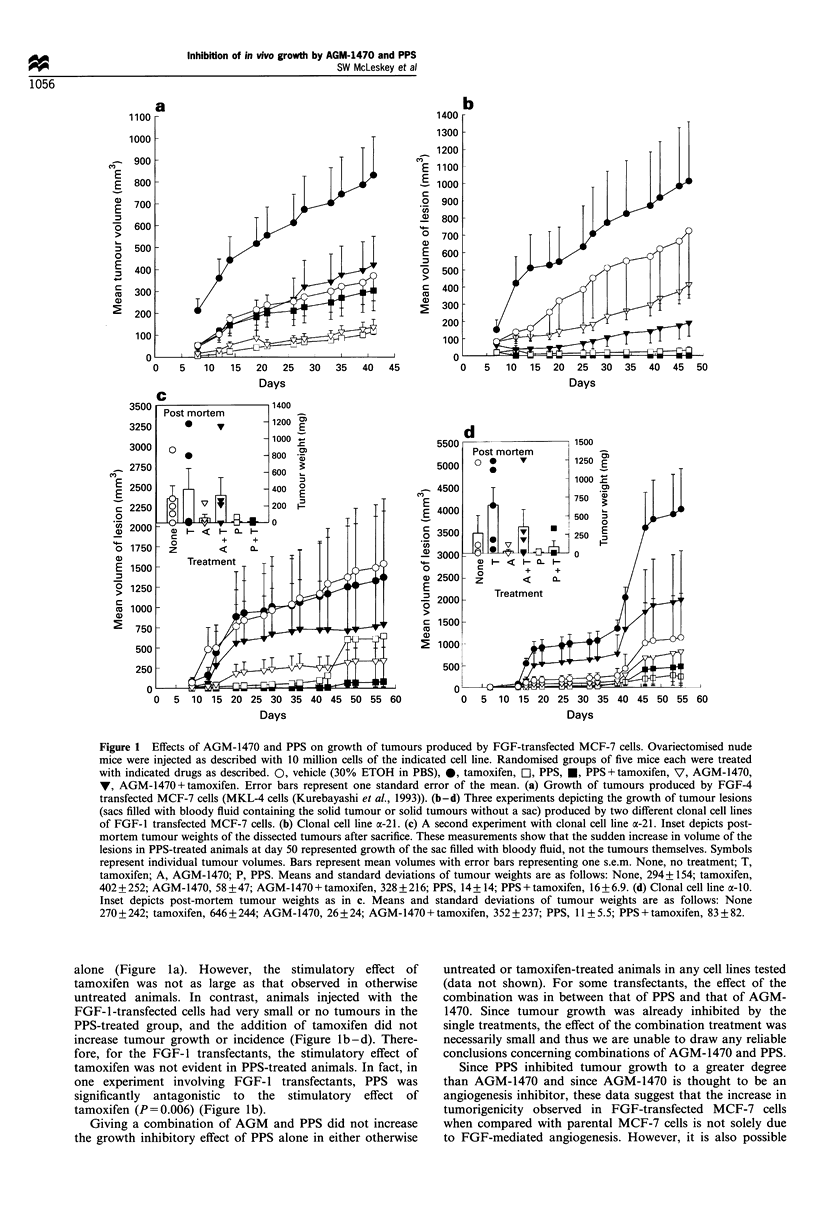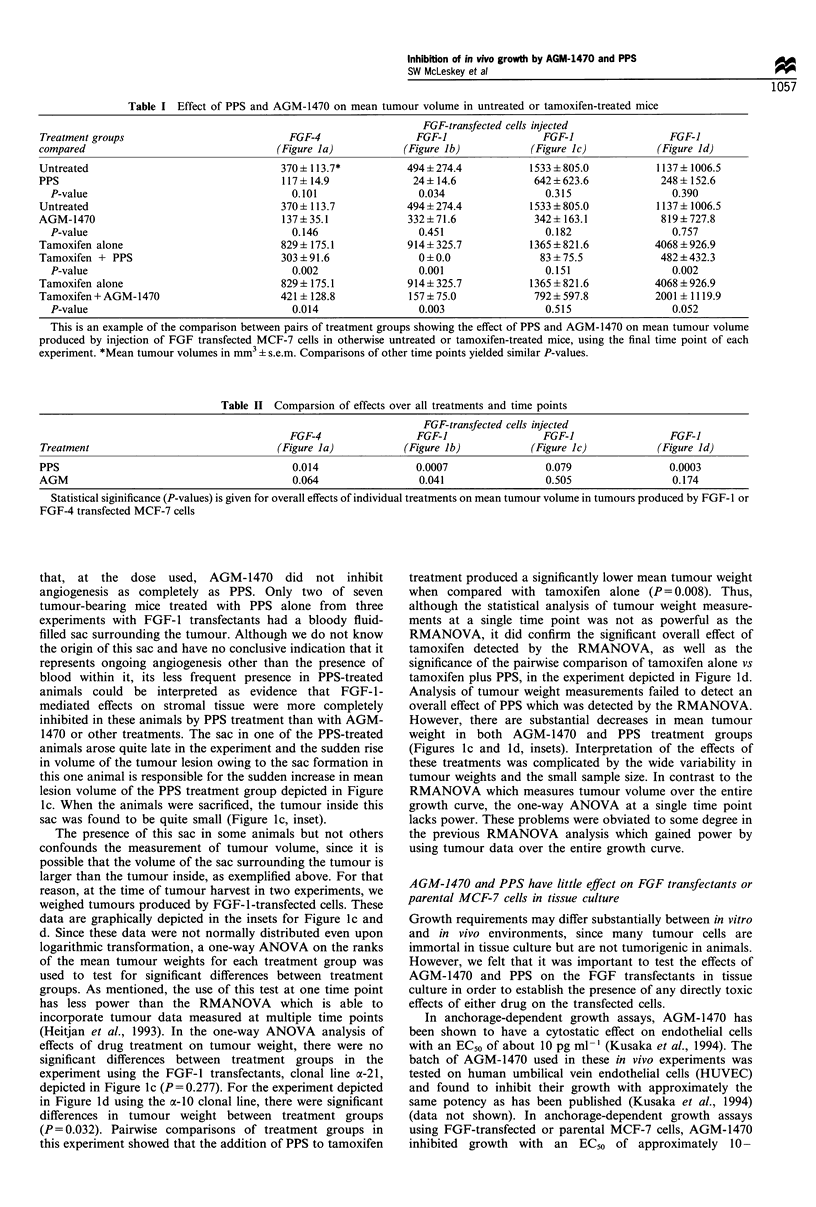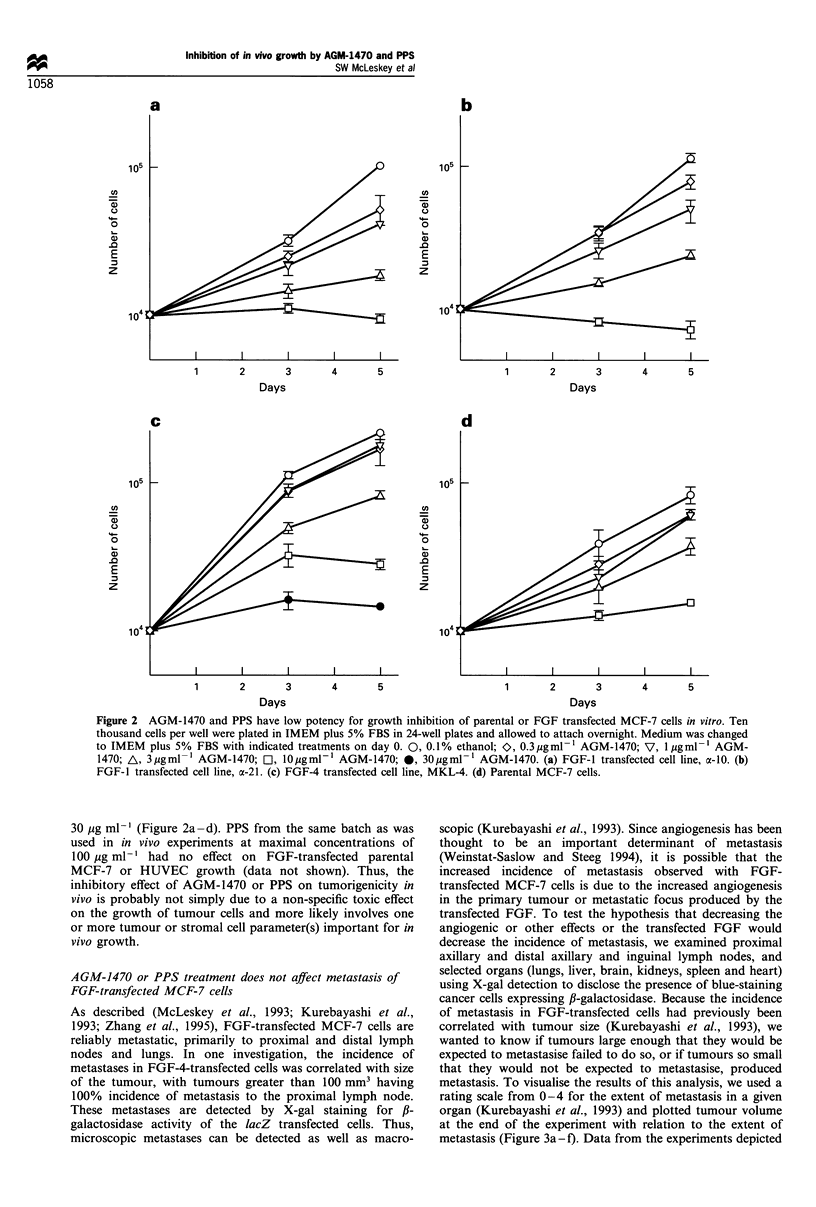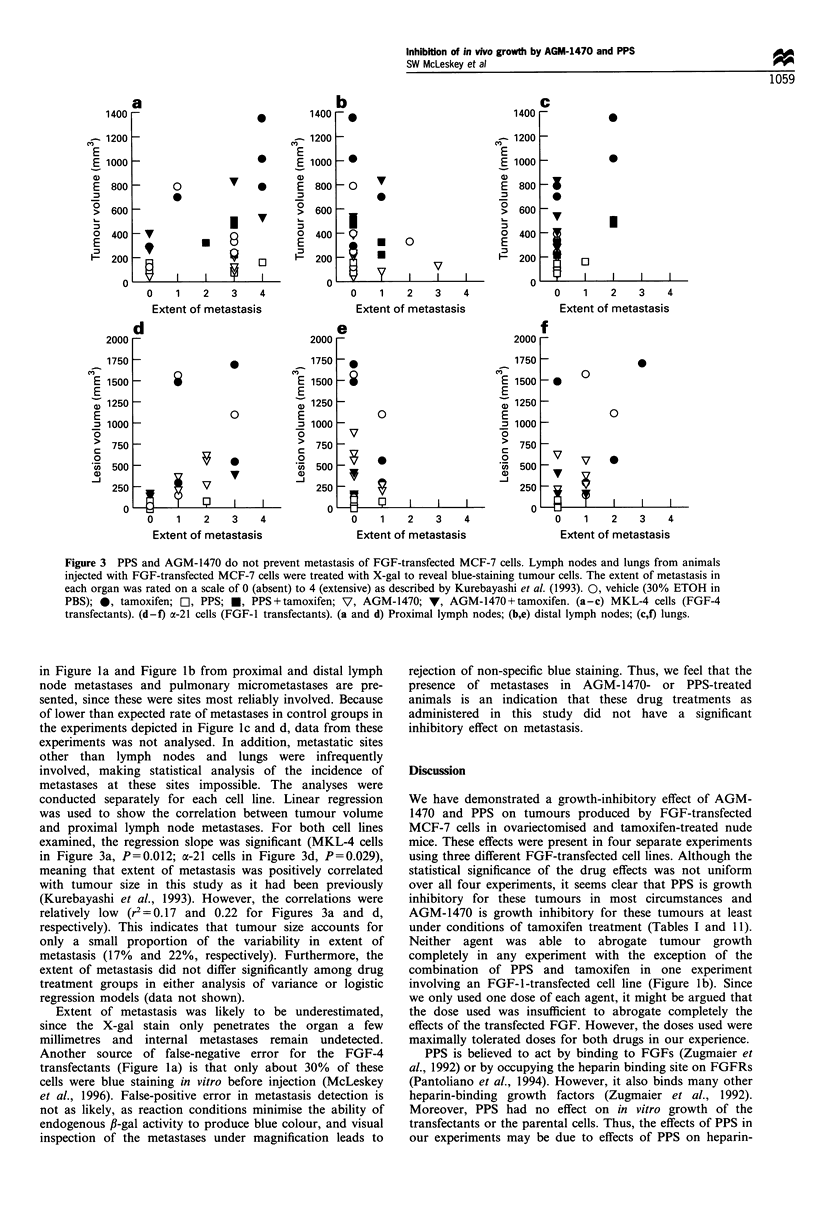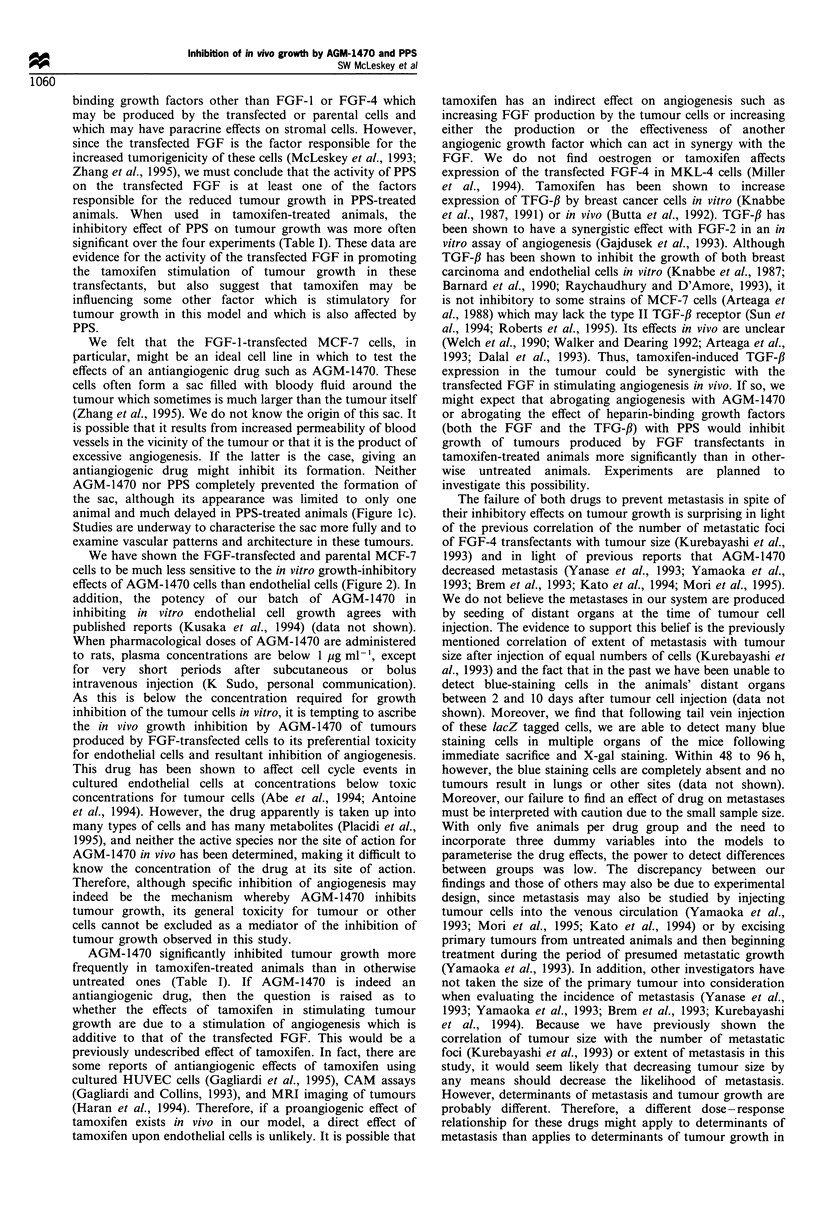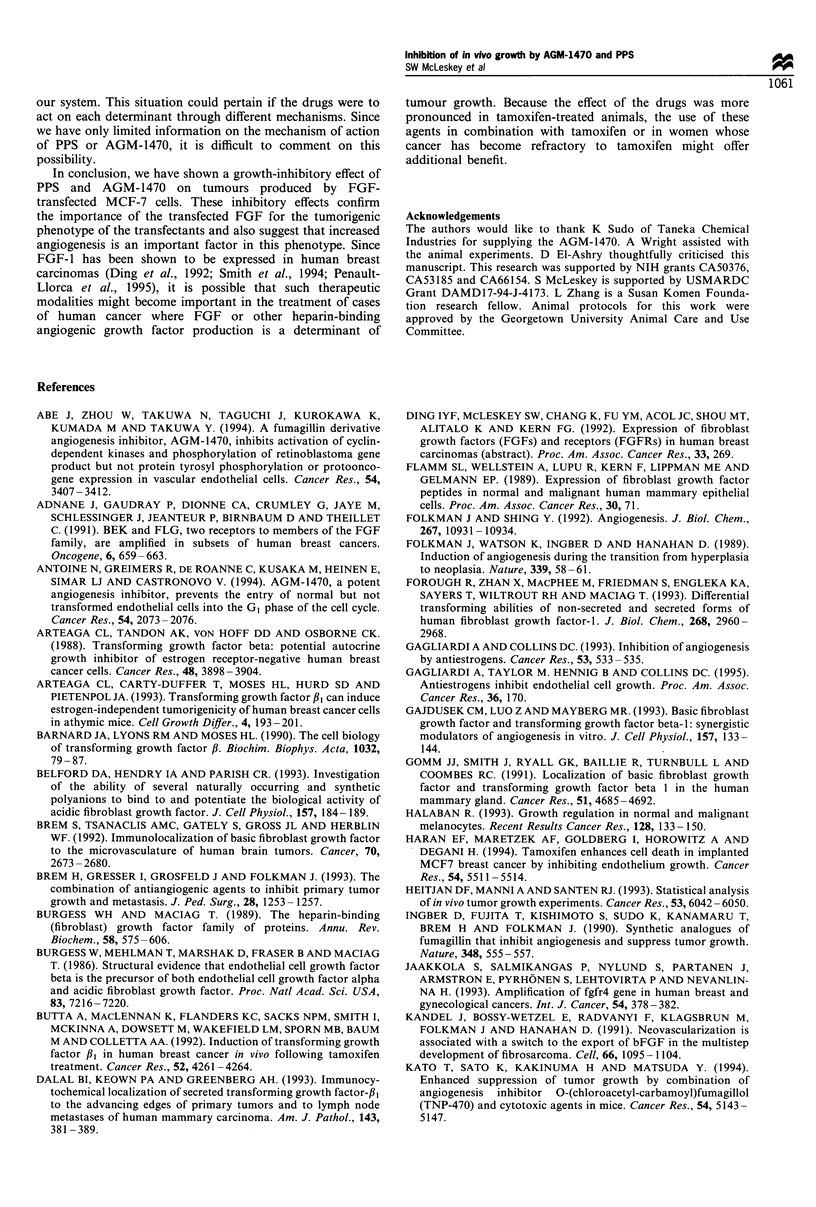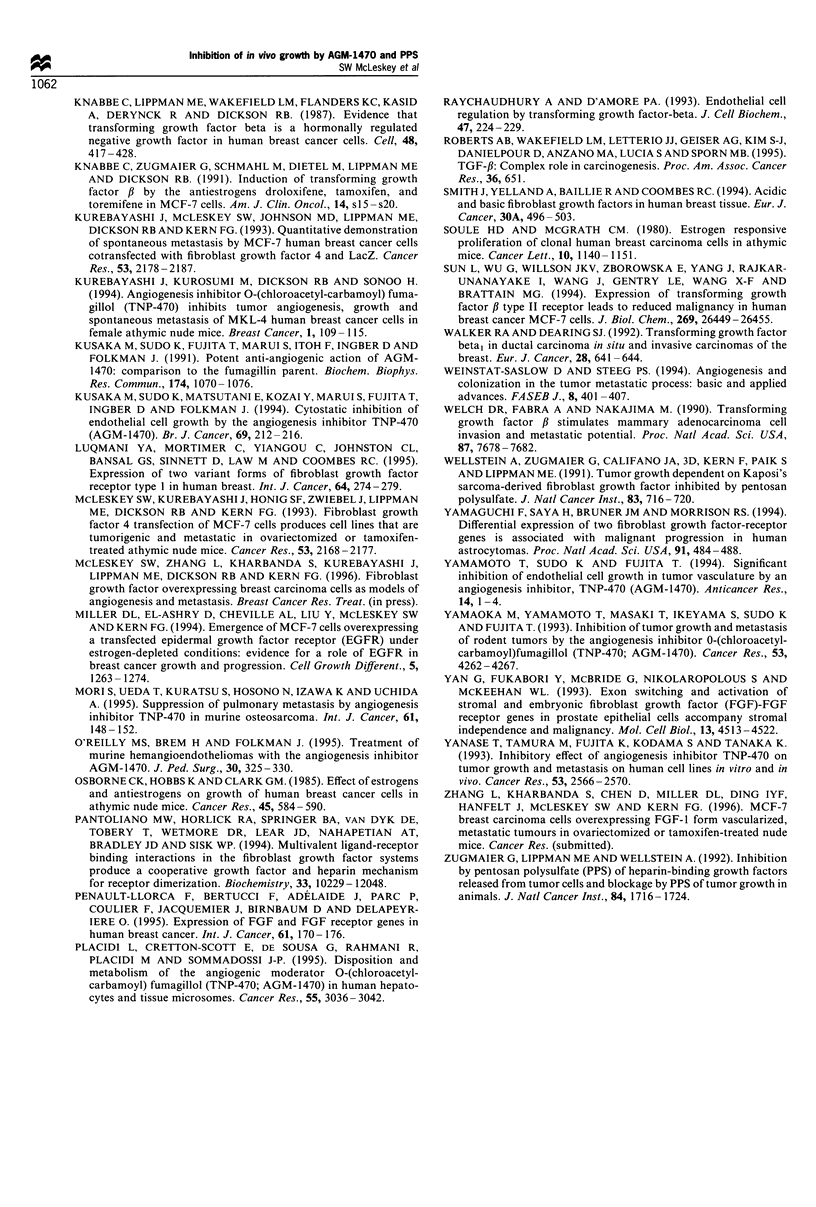# Effects of AGM-1470 and pentosan polysulphate on tumorigenicity and metastasis of FGF-transfected MCF-7 cells.

**DOI:** 10.1038/bjc.1996.204

**Published:** 1996-05

**Authors:** S. W. McLeskey, L. Zhang, B. J. Trock, S. Kharbanda, Y. Liu, M. M. Gottardis, M. E. Lippman, F. G. Kern

**Affiliations:** Lombardi Cancer Center, Georgetown University Medical Center, Washington, DC 20007, USA.

## Abstract

Previously, we described FGF-1- or FGF-4-transfected MCF-7 breast carcinoma cells which are tumorigenic and metastatic in untreated or tamoxifen-treated ovariectomised nude mice. In this study, we have assessed the effects of AGM-1470, an antiangiogenic agent, and pentosan polysulphate (PPS), an agent that abrogates the effects of FGFs, on tumour growth and metastasis produced by these FGF-transfected MCF-7 cells. Untreated or tamoxifen-treated ovariectomised mice were injected with FGF-transfected cells, treated with AGM-1470 or PPS, and tumour growth and metastasis analysed. The sensitivity of FGF-transfected and parental MCF-7 cells to AGM-1470 or PPS was also determined in vitro. Both AGM-1470 and PPS inhibited tumour growth in otherwise untreated or tamoxifen-treated mice injected with either FGF- or FGF-4-transfected MCF-7 cells. This effect was more reliably seen in tamoxifen-treated animals. AGM-1470 was about 10(5) times less potent in inhibiting the anchorage-dependent growth of parental MCF-7 or FGF-transfected MCF-7 cells than in inhibiting the growth of human umbilical vein endothelial cells. PPS did not affect the in vitro growth of the transfectants or parental cells. Thus, the growth-inhibitory effect on tumours was in excess of the effect of either drug on the same cells in tissue culture, implying that stromal elements are important determinants of the effects of these drugs. There was a positive correlation between tumour size and the extent of proximal lymph node metastasis. However, neither drug had a significant effect on the extent of metastasis to proximal or distal lymph nodes or lungs. AGM-1470 or PPS may be helpful in cases of breast carcinoma in which angiogenesis is due to expression of FGFs by the tumour cells and may be more effective when combined with tamoxifen.


British Journal of Cancer (1996) 73, 1053-1062
© 1996 Stockton Press All rights reserved 0007-0920/96 $12.00

### Effects of AGM-1470 and pentosan polysulphate on tumorigenicity and metastasis of FGF-transfected MCF-7 cells

SW McLeskey[1,3,5], L Zhang [1,2], BJ Trock[1,4], S Kharbanda[1], Y Liu[1], MM Gottardis[6], ME Lippman[1,3,4] and FG Kern[1,2]

*[1]Lombardi Cancer Center, Departments of [2]Biochemistry, [3]Pharmacology, [4]Medicine, [5]School of Nursing, Georgetown University Medical Center, Washington, DC 20007; [6]Ligand Pharmaceuticals, Inc., La Jolla, CA 92037, USA.*

**Summary** Previously, we described FGF-1- or FGF-4-transfected MCF-7 breast carcinoma cells which are tumorigenic and metastatic in untreated or tamoxifen-treated ovariectomised nude mice. In this study, we have assessed the effects of AGM-1470, an antiangiogenic agent, and pentosan polysulphate (PPS), an agent that abrogates the effects of FGFs, on tumour growth and metastasis produced by these FGF-transfected MCF-7 cells. Untreated or tamoxifen-treated ovariectomised mice were injected with FGF-transfected cells, treated with AGM-1470 or PPS, and tumour growth and metastasis analysed. The sensitivity of FGF-transfected and parental MCF-7 cells to AGM-1470 or PPS was also determined *in vitro*. Both AGM-1470 and PPS inhibited tumour growth in otherwise untreated or tamoxifen-treated mice injected with either FGF- or FGF-4-transfected MCF-7 cells. This effect was more reliably seen in tamoxifen-treated animals. AGM-1470 was about $10^5$ times less potent in inhibiting the anchorage-dependent growth of parental MCF-7 or FGF-transfected MCF-7 cells than in inhibiting the growth of human umbilical vein endothelial cells. PPS did not affect the *in vitro* growth of the transfectants or parental cells. Thus, the growth-inhibitory effect on tumours was in excess of the effect of either drug on the same cells in tissue culture, implying that stromal elements are important determinants of the effects of these drugs. There was a positive correlation between tumour size and the extent of proximal lymph node metastasis. However, neither drug had a significant effect on the extent of metastasis to proximal or distal lymph nodes or lungs. AGM-1470 or PPS may be helpful in cases of breast carcinoma in which angiogenesis is due to expression of FGFs by the tumour cells and may be more effective when combined with tamoxifen.

**Keywords:** AGM-1470; angiogenesis; FGF; pentosan polysulphate; MCF-7 cells; breast cancer

The acquisition of the ability to promote neovascularisation has been described as a seminal event in tumorigenicity, enabling uncontrolled growth, invasion and metastasis of a previously indolent lesion (Folkman *et al.*, 1989, Folkman Shing, 1992; Weinstat-Saslow and Steeg, 1994). We have previously transfected MCF-7 breast carcinoma cells with expression vectors for one of two angiogenic growth factors, FGF-4 (McLeskey *et al.*, 1993) or FGF-1 (Zhang *et al.*, 1995). These transfections have produced cell lines that are tumorigenic and metastatic in ovariectomised and tamoxifen-treated athymic nude mice. This behaviour is in direct contrast to parental MCF-7 cells, which require oestrogen supplementation for formation of small, poorly metastatic tumours in ovariectomised nude mice (Soule and McGrath, 1980; Osborne *et al.*, 1985). The change in *in vivo* phenotype produced by these transfections may mimic the transition that occurs in oestrogen receptor-positive human breast carcinomas which are initially responsive to antioestrogen therapy. After prolonged antioestrogen therapy, such carcinomas may become refractory to the antioestrogen and acquire a more invasive and metastatic phenotype, ultimately leading to the death of the patient.

Although the acquisition of angiogenic ability may be multifactorial in a given tumour and may involve different mechanisms in different tumours, there is evidence that FGFs or FGF receptors may be involved in some transitions of tumours to an angiogenic phenotype. Expression of FGFs has been associated with a switch to an angiogenic phenotype in fibrosarcomas (Kandel *et al.*, 1991), and is prominent in melanomas (Halaban, 1993) and in brain tumours which are very vascular (Brem *et al.*, 1992). Several investigators have shown specific FGF receptors to be newly expressed at a time of phenotypic transition of tumours from indolent to aggressive or metastatic (Yan *et al.*, 1993; Yamaguchi *et al.*, 1994; Luqmani *et al.*, 1995; Smith *et al.*, 1994; Penault-Llorca *et al.*, 1995; Gomm *et al.*, 1991). We have found FGF-1 or FGF-2 mRNA to be expressed in many breast carcinoma specimens (Ding *et al.*, 1992) and FGF ligands to be preferentially expressed by oestrogen receptor-negative breast carcinoma cell lines (Flamm *et al.*, 1989). Amplified and/or overexpressed FGF receptors have been identified in breast carcinoma specimens, implying that FGF signalling contributes to the phenotype of these tumours (Adnane *et al.*, 1991; Jaakkola *et al.*, 1993).

Since the transfection of FGFs into MCF-7 cells, an oestrogen-dependent, poorly tumorigenic and relatively non-metastatic breast carcinoma cell line, has produced cell lines which cause aggressive, metastasising tumours in the absence of oestrogenic growth stimulation, it seemed important to test the hypothesis that this phenotypic change is the result of increased angiogenesis. Therefore, we have treated ovariectomised mice injected with either FGF-1- or FGF-4-transfected MCF-7 cells with AGM-1470 (also known as TNP470), an antiangiogenic drug (Ingber *et al.*, 1990; Kusaka *et al.*, 1991; Yamamoto *et al.*, 1994; O'Reilly *et al.*, 1995), or pentosan polysulphate (PPS), a drug which binds FGFs as well as other heparin-binding growth factors (Belford *et al.*, 1993) and has been shown to bind to FGFR-1 (Pantoliano *et al.*, 1994), and which under some circumstances inhibits the effects of FGFs (Wellstein *et al.*, 1991; Zugmaier *et al.*, 1992). We now report that both agents were growth inhibitory to tumours produced by FGF-transfected cells in both ovariectomised and tamoxifen-treated mice. These effects were in excess of the *in vitro* effects of these agents on the transfected and parental cells. In spite of the negative effect of each drug on tumour growth and contrary to published reports of an inhibitory effect of AGM-1470 on metastasis in

Correspondence: FG Kern, E416, Research Building, Lombardi Cancer Center, Georgetown University Medical Center, Washington, DC 20007, USA
Received 14 August 1995; revised 8 December 1995; accepted 11 December 1995

other systems (Yanase *et al*., 1993; Yamaoka *et al*., 1993; Brem *et al*., 1993; Mori *et al*., 1995; Kato *et al*., 1994; Kurebayashi *et al*., 1994), neither drug had a detectable inhibitory effect on metastasis in this system.

### Methods

### *Cell lines*

MKL-4 cells are MCF-7 cells sequentially transfected with expression vectors for FGF-4 and *lacZ* as described (McLeskey *et al*., 1993; Kurebayashi *et al*., 1993). α-21 and α-10 cells are clonal G-418-resistant cell lines isolated from a transfection of ML-20 cells [MCF-7 cells first transfected with an expression vector for *lacZ* (Kurebayashi *et al*., 1993)] with an expression vector encoding amino acids 21–145 of FGF-1 (Burgess *et al*., 1986; Burgess and Maciag, 1989), and further characterised as producing high levels of the transfected protein and forming tumours in nude mice (Zhang *et al*., 1995). MCF-7 cells were approximately passage 60.

### *Drugs*

Pentosan polysulphate (PPS) was obtained from beneChemie, Munich, Germany. AGM-1740 (also known as TNP 470) was kindly supplied by Katsuichi Sudo, Takeda Chemical Industries, Osaka, Japan. Tamoxifen pellets (5 mg, 60 day release) were obtained from Innovative Research, Toledo, OH, USA.

### *Cell culture and injection of mice*

Cells were maintained in improved minimal essential medium (IMEM) supplemented with 5% fetal bovine serum (FBS) in a 5% carbon dioxide, 37°C incubator. On the day of injection, cells were scraped into their normal growth medium and viable cells were counted using trypan blue exclusion. Ten million viable cells were injected into the upper right mammary fat pad of each mouse in an injection volume of 0.15 ml. This number of injected cells was used to produce 100% tumour take (McLeskey *et al*., 1993) and is consistent with the numbers of cells injected by others (Haran *et al*., 1994). Two- to 4-week-old virgin athymic nude mice were ovariectomised approximately 2 weeks before each experiment. At the beginning of the experiment, the mice were approximately four- to six-weeks-old and weighed approximately 20 g. Mice were randomised into groups of five and sustained-release tamoxifen pellets (Innovative Research, Toledo, OH, USA) were implanted in the interscapular area as described (McLeskey *et al*., 1993) for half of the groups. Drug treatments were begun the following day. PPS was injected intraperitoneally at a dose of 5 mg $kg^{-1}$ in 0.1 ml phosphate-buffered saline (PBS) 6 days per week. AGM-1470 was injected subcutaneously at a dose of 30 mg $kg^{-1}$ in 0.1 ml of 30% ethanol in PBS three times per week. The control group received subcutaneous 0.1 ml injections of 30% ethanol in PBS. All agents were administered for the duration of the study. Tumours were measured in three dimensions twice weekly with calipers. Tumour volume was calculated as the product of the largest dimension, the orthogonal measurement and tumour thickness. For some experiments, dissected tumours were weighed at the time of sacrifice.

### In vitro *growth curves*

Ten thousand MCF-7 and FGF-transfected MCF-7 cells per well were plated in IMEM with 5% FBS in 24-well plates and allowed to attach overnight. Treatments as indicated in a final volume of 1 ml were added on the following day (day 0). Untreated wells received the ethanol vehicle of AGM-1470 (0.1%). Cells were harvested with 0.1 mM EDTA in PBS on appropriate days and counted using a Coulter automated cell counter. Human umbilical vein endothelial cells (HUVEC) were plated in 24-well plates at 10 000 cells per well using their normal growth medium (IMEM supplemented with 10% FBS, 10 ng $ml^{-1}$ FGF-1, and 10 μg $ml^{-1}$ heparin). Drug treatments were added the day following plating. Cells were harvested as above and counted using a haemocytometer.

### *Detection and rating of metastases*

Metastases in proximal axillary and distal axillary and inguinal lymph nodes and selected whole organs (brain, kidneys, liver, spleen, lungs and heart) were harvested, fixed in 2% formaldehyde, 0.2% glyceraldehyde for 2–3 h, and subjected to staining using X-gal (5-bromo-4-chloro-*d*-indoyl-β-D-galactopyranoside) (1 mg $ml^{-1}$ X-gal in 5 mM potassium ferrocyanide, 5 mM potassium ferricyanide, 2 mM magnesium chloride in PBS) overnight at 4°C. Organs were examined under a dissecting microscope and rated for the presence of blue-staining metastases as described (Kurebayashi *et al*., 1993). Metastases were rated according to the following rating system: 0, no visible blue spots; 1+, few diffuse blue spots (less than about 5) or one microscopic focus of a few blue spots; 2+, diffuse blue spots (about 5–15) or several foci of a few blue spots; 3+, many diffuse blue spots (about 15–50) or a barely visible macroscopic focus of blue; 4+, very many blue spots (more than about 50) or a large macroscopic focus of blue (Kurebayashi *et al*., 1993).

### *Statistical analysis of tumour growth*

Only mice which survived until the end of the experiment were included in the statistical analysis. (Three mice expired of unknown causes, not related to tumour burden, during the course of the four experiments.) Mean tumour volume for each treatment group was obtained using the calculated volumes of each tumour, with zeros being used as the volume when no tumour arose in an animal.

Because the data included tumour volume measurements at multiple time points for each animal, and variances differed between treatment groups and over time, repeated measures analysis of variance (RMANOVA) was used to analyse the data (Heitjan *et al*., 1993). This analysis is considerably more powerful than analyses which compare tumour growth at a single time point. Lack of normality was an inconsistent finding and logarithmic transformations did not improve model fit, so the untransformed data were used for all analyses described here.

The effects of PPS and AGM-1470 were evaluated, singly and in combination, in both untreated and tamoxifen-treated mice. A 2×3 factorial design was used, resulting in the following eight treatment groups, in which each study drug occurs in four of the treatment groups: (1) untreated; (2) AGM-1470 alone; (3) PPS alone; (4) AGM-1470+PPS; (5) tamoxifen alone; (6) tamoxifen+AGM-1470; (7) tamoxifen+PPS; (8) tamoxifen+AGM-1470+PPS.

The analyses were conducted in two ways. The first analysis considered the eight groups above as distinct treatment groups and compared mean tumour volume between pairs of treatment groups at successive time points. This analysis also allowed assessment of *interactions* between drugs. Interaction is said to occur when the measured impact of two treatments in combination is either significantly greater or significantly less than the sum of the effects of each treatment given separately. Interaction is a statistical concept which can suggest, but does not prove, biological synergy or antagonism.

However, these pairwise analyses do not use all information on a particular treatment group. For example, the effect of PPS can be evaluated by comparing group 3 *vs* group 1, but that analysis ignores additional information on the effect of PPS derived from comparing group 7 *vs* group 5. Therefore, we undertook an additional analysis which derives the effect of a drug by considering simultaneously all treatment groups that include the drug *vs* all groups that do not include it. This approach essentially provides an overall test of the drug effect based on the maximum of information. This analysis was used to augment the interpretation derived from the first analysis.

Mammary fat pad injection of FGF-1-transfected cells is sometimes associated with formation of a sac surrounding the tumour which contains bloody fluid (Zhang *et al.*, 1995), as discussed in 'Results'. Tumour volume measurements are then possibly confounded by the presence of the fluid-filled sac. Because some of the tumour volume measurements for tumours produced by FGF-1 transfectants included the volume of the sac, we weighed the harvested tumours from two of the experiments using these FGF-1 transfectants. These data were not normally distributed even upon logarithmic transformation. Consequently, a one-way analysis of variance (ANOVA) was used on the ranks of the mean tumour weights for each treatment group. This non-parametric test of significance at one time point does not have the power of the RMANOVA conducted over multiple time points on the tumour volume data described above and thus may fail to detect a difference between treatment groups when one in fact exists (type II error).

We used analysis of covariance to evaluate the drug effects on the metastasis score for proximal, distant and lung metastases, and a total metastasis score which summed all three site-specific scores. This method allowed us to adjust for the effects of tumour volume at the final time point. In addition, we converted the score to a binary variable for metastases present (yes *vs* no), or low *vs* high total metastasis score (0,1 *vs* 2,3,4). Logistic regression was used to evaluate this binary outcome, again adjusting for the effect of final tumour volume. Because of the small number of animals per treatment group, we included only the effects of single drugs *vs* control in the models (e.g. main effect).

### Results

### *Tumours produced by FGF-1- or FGF-4-transfected MCF-7 cells in nude mice are growth-inhibited by treatment with pentosan polysulphate or AGM-1470*

We treated ovariectomised mice injected with FGF-transfected MCF-7 cells with pentosan polysulphate (PPS), an agent which is capable of abrogating the effects of FGFs and other heparin-binding growth factors *in vitro* and *in vivo* (Wellstein *et al.*, 1991; Zugmaier *et al.*, 1992). This agent presumably acts by binding to FGFs (Belford *et al.*, 1993), preventing them from reaching their receptors on tumour or stromal cells. PPS also may bind to the heparin binding site of FGFR (Pantoliano *et al.*, 1994). By either mechanism, PPS would be expected to abrogate both the autocrine and paracrine effects of the transfected FGFs, reverting the cells back toward their parental phenotype. Since FGFs are known angiogenic factors, we also examined the contribution of the angiogenic component to the tumorigenic phenotype of the transfectants by treating ovariectomised mice injected with FGF-transfected cells with AGM-1470. This agent has preferential toxicity for endothelial cells (Kusaka *et al.*, 1994; Antoine *et al.*, 1994) and is an inhibitor of angiogenesis in many assays (Ingber *et al.*, 1990; Kusaka *et al.*, 1991; Yamamoto *et al.*, 1994). Since we have previously shown that tamoxifen treatment of mice injected with FGF-4-transfected MCF-7 cells stimulates tumour growth (McLeskey *et al.*, 1993), we also tested the effects of PPS or AGM-1470 on tumour growth of FGF transfectants in tamoxifen-treated animals. Our rationale was that abrogation of the effect (either angiogenesis alone with AGM-1470 or all autocrine and paracrine effects with PPS) responsible for the change in phenotype of the transfectants would return them to their parental phenotype of being growth inhibited by tamoxifen treatment. Thus, in these experiments, tamoxifen treatment should be considered as a condition affecting tumour growth rather than an anti-tumour treatment.

The FGF-1-transfected MCF-7 cell lines we chose to use in these experiments are transfected with an expression vector encoding FGF-$1_{21-154}$, a biologically active form of FGF-1 that lacks the first 21 amino acids (Burgess *et al.*, 1986; Burgess and Maciag, 1989; Forough *et al.*, 1993). Although this species lacks a signal peptide sequence, FGF-1 is present in media conditioned by the transfectants. FGF-1-transfected cells exhibit many of the same properties as the FGF-4 transfectants. They are tumorigenic in ovariectomised nude mice without oestrogen supplementation and develop micrometastases in the lymph nodes and lungs with high frequency. One phenotype exhibited by FGF-1 transfectants that was not previously seen with FGF-4 transfectants is the appearance of a sac filled with inflammatory exudate, which does not contain tumour cells, surrounding the tumour in some animals. This sac appears in 40–50% of tumours 1–2 weeks after tumour cell injection. As time progresses, the tumour grows to completely fill the sac (Zhang *et al.*, 1995). To avoid possible error associated with tumour measurements which included the volume of the sac in those instances where it developed, we analysed results of experiments using these cells in several ways below. The FGF-4-transfected cell line used here, MKL-4, has been previously described (McLeskey *et al.*, 1993; Kurebayashi *et al.*, 1993).

Tumour growth curves from four experiments, one with FGF-4-transfected cells and three with FGF-1-transfected cells, are depicted in Figure 1a–d. The information from these curves is summarised in Table I, which includes relevant pairwise comparisons of mean tumour volume between treatment groups. For simplicity, this table only shows comparisons measured at the final time point, but the results for the entire curve are similar.

As can be seen from Figure 1a–d and Table I, PPS reduced tumour volume in all experiments, but the effect was larger and of greater statistical significance for tamoxifen-treated than untreated animals. In other words, the decrease in tumour volume for PPS + tamoxifen *vs* tamoxifen alone was greater than the decrease for PPS *vs* untreated control. Table II indicates that the overall decrease in volume due to PPS (i.e. over all time points and all treatment groups) was significant for all but the second experiment involving the α-21 clonal cell line of FGF-1 transfectants (Figure 1c), where a marginally significant ($P = 0.079$) effect was noted. As noted below, tumour volume measurements in this cell line are confounded by the presence of a fluid-filled sac surrounding the tumour. However, when post mortem tumour weights from the α-21 clonal line were compared in the experiment depicted in Figure 1c, PPS treatment also did not produce an overall statistically significant effect (see below).

AGM-1470 also reduced tumour volume in all experiments. However, as shown in Figure 1a–d and Table I, the effect was larger in tamoxifen-treated animals. Table I shows that, for each transfected cell line, the reduction in tumour volume with AGM-1470 was smaller than that achieved with PPS. Table II shows that the overall effect of AGM-1470 was significant only for the α-21 FGF-1-transfected clonal cell line in the first experiment using this line (Figure 1b), while a marginally significant effect ($P = 0.064$) was observed for the FGF-4 transfectants.

Although both PPS and AGM-1470 exhibited larger effects in the presence of tamoxifen, the statistical tests for interactions between each drug and tamoxifen were not significant. This may reflect the large variability in individual tumour volumes, as indicated by some of the large standard errors in Table I and the small number of animals in each treatment group.

Since PPS presumably reduces FGF-mediated effects in a dose-dependent manner, we could therefore hypothesise that, at some dose, PPS would abrogate the effects of FGFs completely, returning the transfectants to their parental phenotype of being growth inhibited by tamoxifen. At the dose used in these experiments, animals treated with PPS and tamoxifen and injected with FGF-4-transfected cells had a larger mean tumour volume than animals treated with PPS

**Figure 1** Effects of AGM-1470 and PPS on growth of tumours produced by FGF-transfected MCF-7 cells. Ovariectomised nude mice were injected as described with 10 million cells of the indicated cell line. Randomised groups of five mice each were treated with indicated drugs as described. ○, vehicle (30% ETOH in PBS), ●, tamoxifen, □, PPS, ■, PPS + tamoxifen, ▽, AGM-1470, ▼, AGM-1470 + tamoxifen. Error bars represent one standard error of the mean. (**a**) Growth of tumours produced by FGF-4 transfected MCF-7 cells (MKL-4 cells (Kurebayashi *et al*., 1993)). (**b**–**d**) Three experiments depicting the growth of tumour lesions (sacs filled with bloody fluid containing the solid tumour or solid tumours without a sac) produced by two different clonal cell lines of FGF-1 transfected MCF-7 cells. (**b**) Clonal cell line α-21. (**c**) A second experiment with clonal cell line α-21. Inset depicts post-mortem tumour weights of the dissected tumours after sacrifice. These measurements show that the sudden increase in volume of the lesions in PPS-treated animals at day 50 represented growth of the sac filled with bloody fluid, not the tumours themselves. Symbols represent individual tumour volumes. Bars represent mean volumes with error bars representing one s.e.m. None, no treatment; T, tamoxifen; A, AGM-1470; P, PPS. Means and standard deviations of tumour weights are as follows: None, 294 ± 154; tamoxifen, 402 ± 252; AGM-1470, 58 ± 47; AGM-1470 + tamoxifen, 328 ± 216; PPS, 14 ± 14; PPS + tamoxifen, 16 ± 6.9. (**d**) Clonal cell line α-10. Inset depicts post-mortem tumour weights as in **c**. Means and standard deviations of tumour weights are as follows: None 270 ± 242; tamoxifen, 646 ± 244; AGM-1470, 26 ± 24; AGM-1470 + tamoxifen, 352 ± 237; PPS, 11 ± 5.5; PPS + tamoxifen, 83 ± 82.

alone (Figure 1a). However, the stimulatory effect of tamoxifen was not as large as that observed in otherwise untreated animals. In contrast, animals injected with the FGF-1-transfected cells had very small or no tumours in the PPS-treated group, and the addition of tamoxifen did not increase tumour growth or incidence (Figure 1b–d). Therefore, for the FGF-1 transfectants, the stimulatory effect of tamoxifen was not evident in PPS-treated animals. In fact, in one experiment involving FGF-1 transfectants, PPS was significantly antagonistic to the stimulatory effect of tamoxifen ($P = 0.006$) (Figure 1b).

Giving a combination of AGM and PPS did not increase the growth inhibitory effect of PPS alone in either otherwise untreated or tamoxifen-treated animals in any cell lines tested (data not shown). For some transfectants, the effect of the combination was in between that of PPS and that of AGM-1470. Since tumour growth was already inhibited by the single treatments, the effect of the combination treatment was necessarily small and thus we are unable to draw any reliable conclusions concerning combinations of AGM-1470 and PPS.

Since PPS inhibited tumour growth to a greater degree than AGM-1470 and since AGM-1470 is thought to be an angiogenesis inhibitor, these data suggest that the increase in tumorigenicity observed in FGF-transfected MCF-7 cells when compared with parental MCF-7 cells is not solely due to FGF-mediated angiogenesis. However, it is also possible

**Table I** Effect of PPS and AGM-1470 on mean tumour volume in untreated or tamoxifen-treated mice

| *Treatment groups compared* | *FGF-transfected cells injected* FGF-4 (Figure 1a) | FGF-1 (Figure 1b) | FGF-1 (Figure 1c) | FGF-1 (Figure 1d) |
|---|---|---|---|---|
| Untreated | 370±113.7* | 494±274.4 | 1533±805.0 | 1137±1006.5 |
| PPS | 117±14.9 | 24±14.6 | 642±623.6 | 248±152.6 |
| *P*-value | 0.101 | 0.034 | 0.315 | 0.390 |
| Untreated | 370±113.7 | 494±274.4 | 1533±805.0 | 1137±1006.5 |
| AGM-1470 | 137±35.1 | 332±71.6 | 342±163.1 | 819±727.8 |
| *P*-value | 0.146 | 0.451 | 0.182 | 0.757 |
| Tamoxifen alone | 829±175.1 | 914±325.7 | 1365±821.6 | 4068±926.9 |
| Tamoxifen + PPS | 303±91.6 | 0±0.0 | 83±75.5 | 482±432.3 |
| *P*-value | 0.002 | 0.001 | 0.151 | 0.002 |
| Tamoxifen alone | 829±175.1 | 914±325.7 | 1365±821.6 | 4068±926.9 |
| Tamoxifen+AGM-1470 | 421±128.8 | 157±75.0 | 792±597.8 | 2001±1119.9 |
| *P*-value | 0.014 | 0.003 | 0.515 | 0.052 |

This is an example of the comparison between pairs of treatment groups showing the effect of PPS and AGM-1470 on mean tumour volume produced by injection of FGF transfected MCF-7 cells in otherwise untreated or tamoxifen-treated mice, using the final time point of each experiment. *Mean tumour volumes in $mm^3 \pm$ s.e.m. Comparisons of other time points yielded similar *P*-values.

**Table II** Comparsion of effects over all treatments and time points

| *Treatment* | *FGF-transfected cells injected* FGF-4 (Figure 1a) | FGF-1 (Figure 1b) | FGF-1 (Figure 1c) | FGF-1 (Figure 1d) |
|---|---|---|---|---|
| PPS | 0.014 | 0.0007 | 0.079 | 0.0003 |
| AGM | 0.064 | 0.041 | 0.505 | 0.174 |

Statistical siginificance (*P*-values) is given for overall effects of individual treatments on mean tumour volume in tumours produced by FGF-1 or FGF-4 transfected MCF-7 cells that, at the dose used, AGM-1470 did not inhibit angiogenesis as completely as PPS. Only two of seven tumour-bearing mice treated with PPS alone from three experiments with FGF-1 transfectants had a bloody fluid-filled sac surrounding the tumour. Although we do not know the origin of this sac and have no conclusive indication that it represents ongoing angiogenesis other than the presence of blood within it, its less frequent presence in PPS-treated animals could be interpreted as evidence that FGF-1-mediated effects on stromal tissue were more completely inhibited in these animals by PPS treatment than with AGM-1470 or other treatments. The sac in one of the PPS-treated animals arose quite late in the experiment and the sudden rise in volume of the tumour lesion owing to the sac formation in this one animal is responsible for the sudden increase in mean lesion volume of the PPS treatment group depicted in Figure 1c. When the animals were sacrificed, the tumour inside this sac was found to be quite small (Figure 1c, inset).

The presence of this sac in some animals but not others confounds the measurement of tumour volume, since it is possible that the volume of the sac surrounding the tumour is larger than the tumour inside, as exemplified above. For that reason, at the time of tumour harvest in two experiments, we weighed tumours produced by FGF-1-transfected cells. These data are graphically depicted in the insets for Figure 1c and d. Since these data were not normally distributed even upon logarithmic transformation, a one-way ANOVA on the ranks of the mean tumour weights for each treatment group was used to test for significant differences between treatment groups. As mentioned, the use of this test at one time point has less power than the RMANOVA which is able to incorporate tumour data measured at multiple time points (Heitjan *et al.*, 1993). In the one-way ANOVA analysis of effects of drug treatment on tumour weight, there were no significant differences between treatment groups in the experiment using the FGF-1 transfectants, clonal line α-21, depicted in Figure 1c ($P=0.277$). For the experiment depicted in Figure 1d using the α-10 clonal line, there were significant differences in tumour weight between treatment groups ($P=0.032$). Pairwise comparisons of treatment groups in this experiment showed that the addition of PPS to tamoxifen treatment produced a significantly lower mean tumour weight when compared with tamoxifen alone ($P=0.008$). Thus, although the statistical analysis of tumour weight measurements at a single time point was not as powerful as the RMANOVA, it did confirm the significant overall effect of tamoxifen detected by the RMANOVA, as well as the significance of the pairwise comparison of tamoxifen alone *vs* tamoxifen plus PPS, in the experiment depicted in Figure 1d. Analysis of tumour weight measurements failed to detect an overall effect of PPS which was detected by the RMANOVA. However, there are substantial decreases in mean tumour weight in both AGM-1470 and PPS treatment groups (Figures 1c and 1d, insets). Interpretation of the effects of these treatments was complicated by the wide variability in tumour weights and the small sample size. In contrast to the RMANOVA which measures tumour volume over the entire growth curve, the one-way ANOVA at a single time point lacks power. These problems were obviated to some degree in the previous RMANOVA analysis which gained power by using tumour data over the entire growth curve.

### *AGM-1470 and PPS have little effect on FGF transfectants or parental MCF-7 cells in tissue culture*

Growth requirements may differ substantially between *in vitro* and *in vivo* environments, since many tumour cells are immortal in tissue culture but are not tumorigenic in animals. However, we felt that it was important to test the effects of AGM-1470 and PPS on the FGF transfectants in tissue culture in order to establish the presence of any directly toxic effects of either drug on the transfected cells.

In anchorage-dependent growth assays, AGM-1470 has been shown to have a cytostatic effect on endothelial cells with an $EC_{50}$ of about 10 pg $ml^{-1}$ (Kusaka *et al.*, 1994). The batch of AGM-1470 used in these *in vivo* experiments was tested on human umbilical vein endothelial cells (HUVEC) and found to inhibit their growth with approximately the same potency as has been published (Kusaka *et al.*, 1994) (data not shown). In anchorage-dependent growth assays using FGF-transfected or parental MCF-7 cells, AGM-1470 inhibited growth with an $EC_{50}$ of approximately 10–

**Figure 2** AGM-1470 and PPS have low potency for growth inhibition of parental or FGF transfected MCF-7 cells *in vitro*. Ten thousand cells per well were plated in IMEM plus 5% FBS in 24-well plates and allowed to attach overnight. Medium was changed to IMEM plus 5% FBS with indicated treatments on day 0. ○, 0.1% ethanol; ◇, 0.3 $\mu$g ml$^{-1}$ AGM-1470; ▽, 1 $\mu$g ml$^{-1}$ AGM-1470; △, 3 $\mu$g ml$^{-1}$ AGM-1470; □, 10 $\mu$g ml$^{-1}$ AGM-1470; ●, 30 $\mu$g ml$^{-1}$ AGM-1470. (**a**) FGF-1 transfected cell line, $\alpha$-10. (**b**) FGF-1 transfected cell line, $\alpha$-21. (**c**) FGF-4 transfected cell line, MKL-4. (**d**) Parental MCF-7 cells.

30 $\mu$g ml$^{-1}$ (Figure 2a–d). PPS from the same batch as was used in *in vivo* experiments at maximal concentrations of 100 $\mu$g ml$^{-1}$ had no effect on FGF-transfected parental MCF-7 or HUVEC growth (data not shown). Thus, the inhibitory effect of AGM-1470 or PPS on tumorigenicity *in vivo* is probably not simply due to a non-specific toxic effect on the growth of tumour cells and more likely involves one or more tumour or stromal cell parameter(s) important for *in vivo* growth.

### *AGM-1470 or PPS treatment does not affect metastasis of FGF-transfected MCF-7 cells*

As described (McLeskey *et al.*, 1993; Kurebayashi *et al.*, 1993; Zhang *et al.*, 1995), FGF-transfected MCF-7 cells are reliably metastatic, primarily to proximal and distal lymph nodes and lungs. In one investigation, the incidence of metastases in FGF-4-transfected cells was correlated with size of the tumour, with tumours greater than 100 mm$^3$ having 100% incidence of metastasis to the proximal lymph node. These metastases are detected by X-gal staining for $\beta$-galactosidase activity of the *lacZ* transfected cells. Thus, microscopic metastases can be detected as well as macroscopic (Kurebayashi *et al.*, 1993). Since angiogenesis has been thought to be an important determinant of metastasis (Weinstat-Saslow and Steeg 1994), it is possible that the increased incidence of metastasis observed with FGF-transfected MCF-7 cells is due to the increased angiogenesis in the primary tumour or metastatic focus produced by the transfected FGF. To test the hypothesis that decreasing the angiogenic or other effects or the transfected FGF would decrease the incidence of metastasis, we examined proximal axillary and distal axillary and inguinal lymph nodes, and selected organs (lungs, liver, brain, kidneys, spleen and heart) using X-gal detection to disclose the presence of blue-staining cancer cells expressing $\beta$-galactosidase. Because the incidence of metastasis in FGF-transfected cells had previously been correlated with tumour size (Kurebayashi *et al.*, 1993), we wanted to know if tumours large enough that they would be expected to metastasise failed to do so, or if tumours so small that they would not be expected to metastasise, produced metastasis. To visualise the results of this analysis, we used a rating scale from 0–4 for the extent of metastasis in a given organ (Kurebayashi *et al.*, 1993) and plotted tumour volume at the end of the experiment with relation to the extent of metastasis (Figure 3a–f). Data from the experiments depicted

**Figure 3** PPS and AGM-1470 do not prevent metastasis of FGF-transfected MCF-7 cells. Lymph nodes and lungs from animals injected with FGF-transfected MCF-7 cells were treated with X-gal to reveal blue-staining tumour cells. The extent of metastasis in each organ was rated on a scale of 0 (absent) to 4 (extensive) as described by Kurebayashi *et al.* (1993). ○, vehicle (30% ETOH in PBS); ●, tamoxifen; □, PPS; ■, PPS+tamoxifen; ▽, AGM-1470; ▼, AGM-1470+tamoxifen. (**a**–**c**) MKL-4 cells (FGF-4 transfectants). (**d**–**f**) α-21 cells (FGF-1 transfectants). (**a** and **d**) Proximal lymph nodes; (**b**,**e**) distal lymph nodes; (**c**,**f**) lungs.

in Figure 1a and Figure 1b from proximal and distal lymph node metastases and pulmonary micrometastases are presented, since these were sites most reliably involved. Because of lower than expected rate of metastases in control groups in the experiments depicted in Figure 1c and d, data from these experiments was not analysed. In addition, metastatic sites other than lymph nodes and lungs were infrequently involved, making statistical analysis of the incidence of metastases at these sites impossible. The analyses were conducted separately for each cell line. Linear regression was used to show the correlation between tumour volume and proximal lymph node metastases. For both cell lines examined, the regression slope was significant (MKL-4 cells in Figure 3a, $P=0.012$; α-21 cells in Figure 3d, $P=0.029$), meaning that extent of metastasis was positively correlated with tumour size in this study as it had been previously (Kurebayashi *et al.*, 1993). However, the correlations were relatively low ($r^2=0.17$ and 0.22 for Figures 3a and d, respectively). This indicates that tumour size accounts for only a small proportion of the variability in extent of metastasis (17% and 22%, respectively). Furthermore, the extent of metastasis did not differ significantly among drug treatment groups in either analysis of variance or logistic regression models (data not shown).

Extent of metastasis was likely to be underestimated, since the X-gal stain only penetrates the organ a few millimetres and internal metastases remain undetected. Another source of false-negative error for the FGF-4 transfectants (Figure 1a) is that only about 30% of these cells were blue staining *in vitro* before injection (McLeskey *et al.*, 1996). False-positive error in metastasis detection is not as likely, as reaction conditions minimise the ability of endogenous β-gal activity to produce blue colour, and visual inspection of the metastases under magnification leads to rejection of non-specific blue staining. Thus, we feel that the presence of metastases in AGM-1470- or PPS-treated animals is an indication that these drug treatments as administered in this study did not have a significant inhibitory effect on metastasis.

### Discussion

We have demonstrated a growth-inhibitory effect of AGM-1470 and PPS on tumours produced by FGF-transfected MCF-7 cells in ovariectomised and tamoxifen-treated nude mice. These effects were present in four separate experiments using three different FGF-transfected cell lines. Although the statistical significance of the drug effects was not uniform over all four experiments, it seems clear that PPS is growth inhibitory for these tumours in most circumstances and AGM-1470 is growth inhibitory for these tumours at least under conditions of tamoxifen treatment (Tables I and 11). Neither agent was able to abrogate tumour growth completely in any experiment with the exception of the combination of PPS and tamoxifen in one experiment involving an FGF-1-transfected cell line (Figure 1b). Since we only used one dose of each agent, it might be argued that the dose used was insufficient to abrogate completely the effects of the transfected FGF. However, the doses used were maximally tolerated doses for both drugs in our experience.

PPS is believed to act by binding to FGFs (Zugmaier *et al.*, 1992) or by occupying the heparin binding site on FGFRs (Pantoliano *et al.*, 1994). However, it also binds many other heparin-binding growth factors (Zugmaier *et al.*, 1992). Moreover, PPS had no effect on *in vitro* growth of the transfectants or the parental cells. Thus, the effects of PPS in our experiments may be due to effects of PPS on heparinbinding growth factors other than FGF-1 or FGF-4 which may be produced by the transfected or parental cells and which may have paracrine effects on stromal cells. However, since the transfected FGF is the factor responsible for the increased tumorigenicity of these cells (McLeskey *et al*., 1993; Zhang *et al*., 1995), we must conclude that the activity of PPS on the transfected FGF is at least one of the factors responsible for the reduced tumour growth in PPS-treated animals. When used in tamoxifen-treated animals, the inhibitory effect of PPS on tumour growth was more often significant over the four experiments (Table I). These data are evidence for the activity of the transfected FGF in promoting the tamoxifen stimulation of tumour growth in these transfectants, but also suggest that tamoxifen may be influencing some other factor which is stimulatory for tumour growth in this model and which is also affected by PPS.

We felt that the FGF-1-transfected MCF-7 cells, in particular, might be an ideal cell line in which to test the effects of an antiangiogenic drug such as AGM-1470. These cells often form a sac filled with bloody fluid around the tumour which sometimes is much larger than the tumour itself (Zhang *et al*., 1995). We do not know the origin of this sac. It is possible that it results from increased permeability of blood vessels in the vicinity of the tumour or that it is the product of excessive angiogenesis. If the latter is the case, giving an antiangiogenic drug might inhibit its formation. Neither AGM-1470 nor PPS completely prevented the formation of the sac, although its appearance was limited to only one animal and much delayed in PPS-treated animals (Figure 1c). Studies are underway to characterise the sac more fully and to examine vascular patterns and architecture in these tumours.

We have shown the FGF-transfected and parental MCF-7 cells to be much less sensitive to the *in vitro* growth-inhibitory effects of AGM-1470 cells than endothelial cells (Figure 2). In addition, the potency of our batch of AGM-1470 in inhibiting *in vitro* endothelial cell growth agrees with published reports (Kusaka *et al*., 1994) (data not shown). When pharmacological doses of AGM-1470 are administered to rats, plasma concentrations are below 1 $\mu g\ ml^{-1}$, except for very short periods after subcutaneous or bolus intravenous injection (K Sudo, personal communication). As this is below the concentration required for growth inhibition of the tumour cells *in vitro*, it is tempting to ascribe the *in vivo* growth inhibition by AGM-1470 of tumours produced by FGF-transfected cells to its preferential toxicity for endothelial cells and resultant inhibition of angiogenesis. This drug has been shown to affect cell cycle events in cultured endothelial cells at concentrations below toxic concentrations for tumour cells (Abe *et al*., 1994; Antoine *et al*., 1994). However, the drug apparently is taken up into many types of cells and has many metabolites (Placidi *et al*., 1995), and neither the active species nor the site of action for AGM-1470 *in vivo* has been determined, making it difficult to know the concentration of the drug at its site of action. Therefore, although specific inhibition of angiogenesis may indeed be the mechanism whereby AGM-1470 inhibits tumour growth, its general toxicity for tumour or other cells cannot be excluded as a mediator of the inhibition of tumour growth observed in this study.

AGM-1470 significantly inhibited tumour growth more frequently in tamoxifen-treated animals than in otherwise untreated ones (Table I). If AGM-1470 is indeed an antiangiogenic drug, then the question is raised as to whether the effects of tamoxifen in stimulating tumour growth are due to a stimulation of angiogenesis which is additive to that of the transfected FGF. This would be a previously undescribed effect of tamoxifen. In fact, there are some reports of antiangiogenic effects of tamoxifen using cultured HUVEC cells (Gagliardi *et al*., 1995), CAM assays (Gagliardi and Collins, 1993), and MRI imaging of tumours (Haran *et al*., 1994). Therefore, if a proangiogenic effect of tamoxifen exists *in vivo* in our model, a direct effect of tamoxifen upon endothelial cells is unlikely. It is possible that tamoxifen has an indirect effect on angiogenesis such as increasing FGF production by the tumour cells or increasing either the production or the effectiveness of another angiogenic growth factor which can act in synergy with the FGF. We do not find oestrogen or tamoxifen affects expression of the transfected FGF-4 in MKL-4 cells (Miller *et al*., 1994). Tamoxifen has been shown to increase expression of TFG-$\beta$ by breast cancer cells *in vitro* (Knabbe *et al*., 1987, 1991) or *in vivo* (Butta *et al*., 1992). TGF-$\beta$ has been shown to have a synergistic effect with FGF-2 in an *in vitro* assay of angiogenesis (Gajdusek *et al*., 1993). Although TGF-$\beta$ has been shown to inhibit the growth of both breast carcinoma and endothelial cells *in vitro* (Knabbe *et al*., 1987; Barnard *et al*., 1990; Raychaudhury and D'Amore, 1993), it is not inhibitory to some strains of MCF-7 cells (Arteaga *et al*., 1988) which may lack the type II TGF-$\beta$ receptor (Sun *et al*., 1994; Roberts *et al*., 1995). Its effects *in vivo* are unclear (Welch *et al*., 1990; Walker and Dearing 1992; Arteaga *et al*., 1993; Dalal *et al*., 1993). Thus, tamoxifen-induced TGF-$\beta$ expression in the tumour could be synergistic with the transfected FGF in stimulating angiogenesis *in vivo*. If so, we might expect that abrogating angiogenesis with AGM-1470 or abrogating the effect of heparin-binding growth factors (both the FGF and the TFG-$\beta$) with PPS would inhibit growth of tumours produced by FGF transfectants in tamoxifen-treated animals more significantly than in otherwise untreated animals. Experiments are planned to investigate this possibility.

The failure of both drugs to prevent metastasis in spite of their inhibitory effects on tumour growth is surprising in light of the previous correlation of the number of metastatic foci of FGF-4 transfectants with tumour size (Kurebayashi *et al*., 1993) and in light of previous reports that AGM-1470 decreased metastasis (Yanase *et al*., 1993; Yamaoka *et al*., 1993; Brem *et al*., 1993; Kato *et al*., 1994; Mori *et al*., 1995). We do not believe the metastases in our system are produced by seeding of distant organs at the time of tumour cell injection. The evidence to support this belief is the previously mentioned correlation of extent of metastasis with tumour size after injection of equal numbers of cells (Kurebayashi *et al*., 1993) and the fact that in the past we have been unable to detect blue-staining cells in the animals' distant organs between 2 and 10 days after tumour cell injection (data not shown). Moreover, we find that following tail vein injection of these *lacZ* tagged cells, we are able to detect many blue staining cells in multiple organs of the mice following immediate sacrifice and X-gal staining. Within 48 to 96 h, however, the blue staining cells are completely absent and no tumours result in lungs or other sites (data not shown). Moreover, our failure to find an effect of drug on metastases must be interpreted with caution due to the small sample size. With only five animals per drug group and the need to incorporate three dummy variables into the models to parameterise the drug effects, the power to detect differences between groups was low. The discrepancy between our findings and those of others may also be due to experimental design, since metastasis may also be studied by injecting tumour cells into the venous circulation (Yamaoka *et al*., 1993; Mori *et al*., 1995; Kato *et al*., 1994) or by excising primary tumours from untreated animals and then beginning treatment during the period of presumed metastatic growth (Yamaoka *et al*., 1993). In addition, other investigators have not taken the size of the primary tumour into consideration when evaluating the incidence of metastasis (Yanase *et al*., 1993; Yamaoka *et al*., 1993; Brem *et al*., 1993; Kurebayashi *et al*., 1994). Because we have previously shown the correlation of tumour size with the number of metastatic foci (Kurebayashi *et al*., 1993) or extent of metastasis in this study, it would seem likely that decreasing tumour size by any means should decrease the likelihood of metastasis. However, determinants of metastasis and tumour growth are probably different. Therefore, a different dose–response relationship for these drugs might apply to determinants of metastasis than applies to determinants of tumour growth in our system. This situation could pertain if the drugs were to act on each determinant through different mechanisms. Since we have only limited information on the mechanism of action of PPS or AGM-1470, it is difficult to comment on this possibility.

In conclusion, we have shown a growth-inhibitory effect of PPS and AGM-1470 on tumours produced by FGF-transfected MCF-7 cells. These inhibitory effects confirm the importance of the transfected FGF for the tumorigenic phenotype of the transfectants and also suggest that increased angiogenesis is an important factor in this phenotype. Since FGF-1 has been shown to be expressed in human breast carcinomas (Ding *et al.*, 1992; Smith *et al.*, 1994; Penault-Llorca *et al.*, 1995), it is possible that such therapeutic modalities might become important in the treatment of cases of human cancer where FGF or other heparin-binding angiogenic growth factor production is a determinant of tumour growth. Because the effect of the drugs was more pronounced in tamoxifen-treated animals, the use of these agents in combination with tamoxifen or in women whose cancer has become refractory to tamoxifen might offer additional benefit.

**Acknowledgements**

The authors would like to thank K Sudo of Taneka Chemical Industries for supplying the AGM-1470. A Wright assisted with the animal experiments. D El-Ashry thoughtfully criticised this manuscript. This research was supported by NIH grants CA50376, CA53185 and CA66154. S McLeskey is supported by USMARDC Grant DAMD17-94-J-4173. L Zhang is a Susan Komen Foundation research fellow. Animal protocols for this work were approved by the Georgetown University Animal Care and Use Committee.

### References

ABE J, ZHOU W, TAKUWA N, TAGUCHI J, KUROKAWA K, KUMADA M AND TAKUWA Y. (1994). A fumagillin derivative angiogenesis inhibitor, AGM-1470, inhibits activation of cyclin-dependent kinases and phosphorylation of retinoblastoma gene product but not protein tyrosyl phosphorylation or protooncogene expression in vascular endothelial cells. *Cancer Res.*, **54,** 3407–3412.

ADNANE J, GAUDRAY P, DIONNE CA, CRUMLEY G, JAYE M, SCHLESSINGER J, JEANTEUR P, BIRNBAUM D AND THEILLET C. (1991). BEK and FLG, two receptors to members of the FGF family, are amplified in subsets of human breast cancers. *Oncogene*, **6,** 659–663.

ANTOINE N, GREIMERS R, DE ROANNE C, KUSAKA M, HEINEN E, SIMAR LJ AND CASTRONOVO V. (1994). AGM-1470, a potent angiogenesis inhibitor, prevents the entry of normal but not transformed endothelial cells into the $G_1$ phase of the cell cycle. *Cancer Res.*, **54,** 2073–2076.

ARTEAGA CL, TANDON AK, VON HOFF DD AND OSBORNE CK. (1988). Transforming growth factor beta: potential autocrine growth inhibitor of estrogen receptor-negative human breast cancer cells. *Cancer Res.*, **48,** 3898–3904.

ARTEAGA CL, CARTY-DUFFER T, MOSES HL, HURD SD AND PIETENPOL JA. (1993). Transforming growth factor $\beta_1$ can induce estrogen-independent tumorigenicity of human breast cancer cells in athymic mice. *Cell Growth Differ.*, **4,** 193–201.

BARNARD JA, LYONS RM AND MOSES HL. (1990). The cell biology of transforming growth factor *β*. *Biochim. Biophys. Acta*, **1032,** 79–87.

BELFORD DA, HENDRY IA AND PARISH CR. (1993). Investigation of the ability of several naturally occurring and synthetic polyanions to bind to and potentiate the biological activity of acidic fibroblast growth factor. *J. Cell Physiol.*, **157,** 184–189.

BREM S, TSANACLIS AMC, GATELY S, GROSS JL AND HERBLIN WF. (1992). Immunolocalization of basic fibroblast growth factor to the microvasculature of human brain tumors. *Cancer*, **70,** 2673–2680.

BREM H, GRESSER I, GROSFELD J AND FOLKMAN J. (1993). The combination of antiangiogenic agents to inhibit primary tumor growth and metastasis. *J. Ped. Surg.*, **28,** 1253–1257.

BURGESS WH AND MACIAG T. (1989). The heparin-binding (fibroblast) growth factor family of proteins. *Annu. Rev. Biochem.*, **58,** 575–606.

BURGESS W, MEHLMAN T, MARSHAK D, FRASER B AND MACIAG T. (1986). Structural evidence that endothelial cell growth factor beta is the precursor of both endothelial cell growth factor alpha and acidic fibroblast growth factor. *Proc. Natl Acad. Sci. USA*, **83,** 7216–7220.

BUTTA A, MACLENNAN K, FLANDERS KC, SACKS NPM, SMITH I, MCKINNA A, DOWSETT M, WAKEFIELD LM, SPORN MB, BAUM M AND COLLETTA AA. (1992). Induction of transforming growth factor $\beta_1$ in human breast cancer *in vivo* following tamoxifen treatment. *Cancer Res.*, **52,** 4261–4264.

DALAL BI, KEOWN PA AND GREENBERG AH. (1993). Immunocytochemical localization of secreted transforming growth factor-$\beta_1$ to the advancing edges of primary tumors and to lymph node metastases of human mammary carcinoma. *Am. J. Pathol.*, **143,** 381–389.

DING IYF, MCLESKEY SW, CHANG K, FU YM, ACOL JC, SHOU MT, ALITALO K AND KERN FG. (1992). Expression of fibroblast growth factors (FGFs) and receptors (FGFRs) in human breast carcinomas (abstract). *Proc. Am. Assoc. Cancer Res.*, **33,** 269.

FLAMM SL, WELLSTEIN A, LUPU R, KERN F, LIPPMAN ME AND GELMANN EP. (1989). Expression of fibroblast growth factor peptides in normal and malignant human mammary epithelial cells. *Proc. Am. Assoc. Cancer Res.*, **30,** 71.

FOLKMAN J AND SHING Y. (1992). Angiogenesis. *J. Biol. Chem.*, **267,** 10931–10934.

FOLKMAN J, WATSON K, INGBER D AND HANAHAN D. (1989). Induction of angiogenesis during the transition from hyperplasia to neoplasia. *Nature*, **339,** 58–61.

FOROUGH R, ZHAN X, MACPHEE M, FRIEDMAN S, ENGLEKA KA, SAYERS T, WILTROUT RH AND MACIAG T. (1993). Differential transforming abilities of non-secreted and secreted forms of human fibroblast growth factor-1. *J. Biol. Chem.*, **268,** 2960–2968.

GAGLIARDI A AND COLLINS DC. (1993). Inhibition of angiogenesis by antiestrogens. *Cancer Res.*, **53,** 533–535.

GAGLIARDI A, TAYLOR M. HENNIG B AND COLLINS DC. (1995). Antiestrogens inhibit endothelial cell growth. *Proc. Am. Assoc. Cancer Res.*, **36,** 170.

GAJDUSEK CM, LUO Z AND MAYBERG MR. (1993). Basic fibroblast growth factor and transforming growth factor beta-1: synergistic modulators of angiogenesis in vitro. *J. Cell Physiol.*, **157,** 133–144.

GOMM JJ, SMITH J, RYALL GK, BAILLIE R, TURNBULL L AND COOMBES RC. (1991). Localization of basic fibroblast growth factor and transforming growth factor beta 1 in the human mammary gland. *Cancer Res.*, **51,** 4685–4692.

HALABAN R. (1993). Growth regulation in normal and malignant melanocytes. *Recent Results Cancer Res.*, **128,** 133–150.

HARAN EF, MARETZEK AF, GOLDBERG I, HOROWITZ A AND DEGANI H. (1994). Tamoxifen enhances cell death in implanted MCF7 breast cancer by inhibiting endothelium growth. *Cancer Res.*, **54,** 5511–5514.

HEITJAN DF, MANNI A AND SANTEN RJ. (1993). Statistical analysis of *in vivo* tumor growth experiments. *Cancer Res.*, **53,** 6042–6050.

INGBER D, FUJITA T, KISHIMOTO S, SUDO K, KANAMARU T, BREM H AND FOLKMAN J. (1990). Synthetic analogues of fumagillin that inhibit angiogenesis and suppress tumor growth. *Nature*, **348,** 555–557.

JAAKKOLA S, SALMIKANGAS P, NYLUND S, PARTANEN J, ARMSTRON E, PYRHÖNEN S, LEHTOVIRTA P AND NEVANLINNA H. (1993). Amplification of fgfr4 gene in human breast and gynecological cancers. *Int. J. Cancer*, **54,** 378–382.

KANDEL J, BOSSY-WETZEL E, RADVANYI F, KLAGSBRUN M, FOLKMAN J AND HANAHAN D. (1991). Neovascularization is associated with a switch to the export of bFGF in the multistep development of fibrosarcoma. *Cell*, **66,** 1095–1104.

KATO T, SATO K, KAKINUMA H AND MATSUDA Y. (1994). Enhanced suppression of tumor growth by combination of angiogenesis inhibitor O-(chloroacetyl-carbamoyl)fumagillol (TNP-470) and cytotoxic agents in mice. *Cancer Res.*, **54,** 5143–5147.

KNABBE C, LIPPMAN ME, WAKEFIELD LM, FLANDERS KC, KASID A, DERYNCK R AND DICKSON RB. (1987). Evidence that transforming growth factor beta is a hormonally regulated negative growth factor in human breast cancer cells. *Cell*, **48**, 417–428.

KNABBE C, ZUGMAIER G, SCHMAHL M, DIETEL M, LIPPMAN ME AND DICKSON RB. (1991). Induction of transforming growth factor $\beta$ by the antiestrogens droloxifene, tamoxifen, and toremifene in MCF-7 cells. *Am. J. Clin. Oncol.*, **14**, s15–s20.

KUREBAYASHI J, MCLESKEY SW, JOHNSON MD, LIPPMAN ME, DICKSON RB AND KERN FG. (1993). Quantitative demonstration of spontaneous metastasis by MCF-7 human breast cancer cells cotransfected with fibroblast growth factor 4 and LacZ. *Cancer Res.*, **53**, 2178–2187.

KUREBAYASHI J, KUROSUMI M, DICKSON RB AND SONOO H. (1994). Angiogenesis inhibitor O-(chloroacetyl-carbamoyl) fumagillol (TNP-470) inhibits tumor angiogenesis, growth and spontaneous metastasis of MKL-4 human breast cancer cells in female athymic nude mice. *Breast Cancer*, **1**, 109–115.

KUSAKA M, SUDO K, FUJITA T, MARUI S, ITOH F, INGBER D AND FOLKMAN J. (1991). Potent anti-angiogenic action of AGM-1470: comparison to the fumagillin parent. *Biochem. Biophys. Res. Commun.*, **174**, 1070–1076.

KUSAKA M, SUDO K, MATSUTANI E, KOZAI Y, MARUI S, FUJITA T, INGBER D AND FOLKMAN J. (1994). Cytostatic inhibition of endothelial cell growth by the angiogenesis inhibitor TNP-470 (AGM-1470). *Br. J. Cancer*, **69**, 212–216.

LUQMANI YA, MORTIMER C, YIANGOU C, JOHNSTON CL, BANSAL GS, SINNETT D, LAW M AND COOMBES RC. (1995). Expression of two variant forms of fibroblast growth factor receptor type 1 in human breast. *Int. J. Cancer*, **64**, 274–279.

MCLESKEY SW, KUREBAYASHI J, HONIG SF, ZWIEBEL J, LIPPMAN ME, DICKSON RB AND KERN FG. (1993). Fibroblast growth factor 4 transfection of MCF-7 cells produces cell lines that are tumorigenic and metastatic in ovariectomized or tamoxifen-treated athymic nude mice. *Cancer Res.*, **53**, 2168–2177.

MCLESKEY SW, ZHANG L, KHARBANDA S, KUREBAYASHI J, LIPPMAN ME, DICKSON RB AND KERN FG. (1996). Fibroblast growth factor overexpressing breast carcinoma cells as models of angiogenesis and metastasis. *Breast Cancer Res. Treat.* (in press).

MILLER DL, EL-ASHRY D, CHEVILLE AL, LIU Y, MCLESKEY SW AND KERN FG. (1994). Emergence of MCF-7 cells overexpressing a transfected epidermal growth factor receptor (EGFR) under estrogen-depleted conditions: evidence for a role of EGFR in breast cancer growth and progression. *Cell Growth Different.*, **5**, 1263–1274.

MORI S, UEDA T, KURATSU S, HOSONO N, IZAWA K AND UCHIDA A. (1995). Suppression of pulmonary metastasis by angiogenesis inhibitor TNP-470 in murine osteosarcoma. *Int. J. Cancer*, **61**, 148–152.

O'REILLY MS, BREM H AND FOLKMAN J. (1995). Treatment of murine hemangioendotheliomas with the angiogenesis inhibitor AGM-1470. *J. Ped. Surg.*, **30**, 325–330.

OSBORNE CK, HOBBS K AND CLARK GM. (1985). Effect of estrogens and antiestrogens on growth of human breast cancer cells in athymic nude mice. *Cancer Res.*, **45**, 584–590.

PANTOLIANO MW, HORLICK RA, SPRINGER BA, VAN DYK DE, TOBERY T, WETMORE DR, LEAR JD, NAHAPETIAN AT, BRADLEY JD AND SISK WP. (1994). Multivalent ligand-receptor binding interactions in the fibroblast growth factor systems produce a cooperative growth factor and heparin mechanism for receptor dimerization. *Biochemistry*, **33**, 10229–12048.

PENAULT-LLORCA F, BERTUCCI F, ADÉLAIDE J, PARC P, COULIER F, JACQUEMIER J, BIRNBAUM D AND DELAPEYRIERE O. (1995). Expression of FGF and FGF receptor genes in human breast cancer. *Int. J. Cancer*, **61**, 170–176.

PLACIDI L, CRETTON-SCOTT E, DE SOUSA G, RAHMANI R, PLACIDI M AND SOMMADOSSI J-P. (1995). Disposition and metabolism of the angiogenic moderator O-(chloroacetyl-carbamoyl) fumagillol (TNP-470; AGM-1470) in human hepatocytes and tissue microsomes. *Cancer Res.*, **55**, 3036–3042.

RAYCHAUDHURY A AND D'AMORE PA. (1993). Endothelial cell regulation by transforming growth factor-beta. *J. Cell Biochem.*, **47**, 224–229.

ROBERTS AB, WAKEFIELD LM, LETTERIO JJ, GEISER AG, KIM S-J, DANIELPOUR D, ANZANO MA, LUCIA S AND SPORN MB. (1995). TGF-$\beta$: Complex role in carcinogenesis. *Proc. Am. Assoc. Cancer Res.*, **36**, 651.

SMITH J, YELLAND A, BAILLIE R AND COOMBES RC. (1994). Acidic and basic fibroblast growth factors in human breast tissue. *Eur. J. Cancer*, **30A**, 496–503.

SOULE HD AND MCGRATH CM. (1980). Estrogen responsive proliferation of clonal human breast carcinoma cells in athymic mice. *Cancer Lett.*, **10**, 1140–1151.

SUN L, WU G, WILLSON JKV, ZBOROWSKA E, YANG J, RAJKARUNANAYAKE I, WANG J, GENTRY LE, WANG X-F AND BRATTAIN MG. (1994). Expression of transforming growth factor $\beta$ type II receptor leads to reduced malignancy in human breast cancer MCF-7 cells. *J. Biol. Chem.*, **269**, 26449–26455.

WALKER RA AND DEARING SJ. (1992). Transforming growth factor $beta_1$ in ductal carcinoma *in situ* and invasive carcinomas of the breast. *Eur. J. Cancer*, **28**, 641–644.

WEINSTAT-SASLOW D AND STEEG PS. (1994). Angiogenesis and colonization in the tumor metastatic process: basic and applied advances. *FASEB J.*, **8**, 401–407.

WELCH DR, FABRA A AND NAKAJIMA M. (1990). Transforming growth factor $\beta$ stimulates mammary adenocarcinoma cell invasion and metastatic potential. *Proc. Natl Acad. Sci. USA*, **87**, 7678–7682.

WELLSTEIN A, ZUGMAIER G, CALIFANO JA, 3D, KERN F, PAIK S AND LIPPMAN ME. (1991). Tumor growth dependent on Kaposi's sarcoma-derived fibroblast growth factor inhibited by pentosan polysulfate. *J. Natl Cancer Inst.*, **83**, 716–720.

YAMAGUCHI F, SAYA H, BRUNER JM AND MORRISON RS. (1994). Differential expression of two fibroblast growth factor-receptor genes is associated with malignant progression in human astrocytomas. *Proc. Natl Acad. Sci. USA*, **91**, 484–488.

YAMAMOTO T, SUDO K AND FUJITA T. (1994). Significant inhibition of endothelial cell growth in tumor vasculature by an angiogenesis inhibitor, TNP-470 (AGM-1470). *Anticancer Res.*, **14**, 1–4.

YAMAOKA M, YAMAMOTO T, MASAKI T, IKEYAMA S, SUDO K AND FUJITA T. (1993). Inhibition of tumor growth and metastasis of rodent tumors by the angiogenesis inhibitor 0-(chloroacetyl-carbamoyl)fumagillol (TNP-470; AGM-1470). *Cancer Res.*, **53**, 4262–4267.

YAN G, FUKABORI Y, MCBRIDE G, NIKOLAROPOLOUS S AND MCKEEHAN WL. (1993). Exon switching and activation of stromal and embryonic fibroblast growth factor (FGF)-FGF receptor genes in prostate epithelial cells accompany stromal independence and malignancy. *Mol. Cell Biol.*, **13**, 4513–4522.

YANASE T, TAMURA M, FUJITA K, KODAMA S AND TANAKA K. (1993). Inhibitory effect of angiogenesis inhibitor TNP-470 on tumor growth and metastasis on human cell lines *in vitro* and *in vivo*. *Cancer Res.*, **53**, 2566–2570.

ZHANG L, KHARBANDA S, CHEN D, MILLER DL, DING IYF, HANFELT J, MCLESKEY SW AND KERN FG. (1996). MCF-7 breast carcinoma cells overexpressing FGF-1 form vascularized, metastatic tumours in ovariectomized or tamoxifen-treated nude mice. *Cancer Res.* (submitted).

ZUGMAIER G, LIPPMAN ME AND WELLSTEIN A. (1992). Inhibition by pentosan polysulfate (PPS) of heparin-binding growth factors released from tumor cells and blockage by PPS of tumor growth in animals. *J. Natl Cancer Inst.*, **84**, 1716–1724.